# Angiosarcoma of bone: a retrospective study of the European Musculoskeletal Oncology Society (EMSOS)

**DOI:** 10.1038/s41598-020-66579-5

**Published:** 2020-07-02

**Authors:** Emanuela Palmerini, Andreas Leithner, Reinhard Windhager, Georg Gosheger, Kjetil Boye, Minna Laitinen, Jendrik Hardes, Frank Traub, Paul Jutte, Madeleine Willegger, Jose’ Casanova, Elisabetta Setola, Alberto Righi, Piero Picci, Davide Maria Donati, Stefano Ferrari

**Affiliations:** 1 IRCCS Istituto Ortopedico Rizzoli, Bologna, Italy; 2 Department of Orthopaedics and Trauma, Medical University of Graz, Graz, Austria; 3 Medical University of Vienna, Department of Orthopaedic Surgery, Vienna, Austria; 4 Westfalian Wilhelms University, University Hospital Muenster, Department of Orthopaedics and Tumor Orthopaedics, Muenster, Germany; 5 Department of Oncology, Oslo University Hospital, Oslo, Norway; 6 Bone Tumour Unit, Department of Orthopaedics and Traumatology Helsinki University Hospital, University of Helsinki, Helsinki, Finland; 7 Department of Orthopaedic Surgery Eberhard Karls University, Tuebingen, Germany; 8 Department of Orthopaedic Surgery, University Medical Center Groningen, University of Groningen, Groningen, the Netherlands; 9 Orthopedic University Hospital, University of Coimbra, Coimbra, Portugal

**Keywords:** Bone cancer, Surgical oncology

## Abstract

Angiosarcoma of bone (B-AS) is a rare malignant tumor of vascular origin. The aim of this retrospective study is to report on treatments and prognosis. Data were collected from the EMSOS website. 80 patients in 9 centers included: 51 male/29 female; median age 54 years (range 17 to 92); 56% with localized disease, 44% metastatic. Primary tumor surgery: 76% (30% amputation, 26% intralesional margins); radiotherapy (RT): 41%; chemotherapy (CT): 47% (56% in metastatic, 41% in localized cases). With a median follow-up of 31 months (range 40 to 309), 5-year overall survival (OS) was 27% (95%CI 16–30): 41% (95%CI 25–56) for localized patients, and 8% (95%CI 0–20) for metastatic (p = 0.002). In metastatic patients, 1 year OS was significantly influenced by chemotherapy response: 67% (95CI% 29–100) for those who responded or had stable disease (n = 7), and 18% (95CI% 0–41) for patients with progressive disease (n = 11), p 0.002. The surgical complete remission (SCR) status was pivotal in localized patients (5-year OS 45% for SCR, 17% no SCR, p = 0.03); also 5-year OS was significantly influenced by age and site of the tumor. After multivariate analysis, the addition of radiotherapy to surgery significantly influenced the disease-free survival (DFS) rate, whereas the use of chemotherapy lost the significance showed at the univariate analysis. Overall, patients with metastatic B-AS have a dismal prognosis, with a prolonged survival in case with a response to chemotherapy. Experimental trials with more active systemic treatment regimens are needed. In patients with localized disease, the patient’s age and site of the tumor are prognostic factors and any effort must be made to achieve an SCR status. No definitive conclusions can be drawn from our data on the use of adjuvant chemotherapy, while the use of adjuvant radiotherapy might improve DSF in patients surgically free of disease.

## Introduction

**Figure 1:**
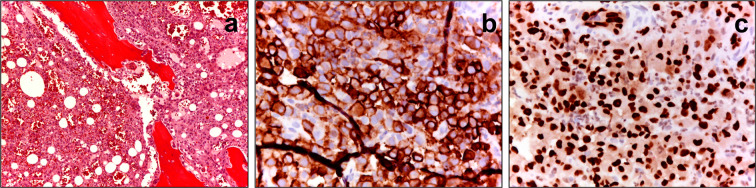
Bone angiosarcoma microscopy: irregular and haphazard blood-filled cavities rimmed by highly malignant atypical cells diffusely permeating the host trabeculae at hematoxilin and eosin staining (panel a), with a strong CD31 (panel b) and ERG (panel c) positivity.

Angiosarcoma of bone (B-AS) is exceedingly rare, accounting for less than 1% of all primary bone sarcomas, with the highest incidence between 50 and 70 years of age^1,4^. The diagnosis of B-AS is challenging and represents the malignant end of the spectrum of CD31/ERG positive vascular tumors, including hemangiomas, hemangioendotheliomas, well-differentiated and poorly differentiated angiosarcomas^1–3^ (Fig. 1). The disease might present as unifocal or with multifocal bone lesions, and it is usually associated with a poor prognosis^1–6^. Given the rarity of B-AS, only scant information can be found in the literature, mostly reported in form of small series and case reports. No specific treatment guidelines or position papers are available, so the treatment of choice is based on data from other types of bone and soft tissue sarcomas^1,3–7^. The role of chemotherapy and prognostic factors for these patients is still unclear^8,9^. Published larger series results are difficult to interpret due to inclusion of both low and high-grade vascular tumors of bone^10–12^, soft tissue and B-AS^13^, or lack of information on treatments^3,14^.

A European retrospective study has been carried out. The European Musculoskeletal Oncology Society (EMSOS) supported the study that was open to the members of the society. Clinical characteristics, treatment modalities and outcome were collected and the final analysis of the data is here reported.

## Patient and Methods

### Patient selection

Through the EMSOS website the study protocol and the data set for the collection of the information of interest were made available to the EMSOS members. Study protocol was approved by the Ethics Committee of the IRCCS Istituto Ortopedico Rizzoli (coordinating center for the study), and informed consent and privacy agreements were obtained from all participants and/or their legal guardians.

All methods were performed in accordance with relevant guidelines and regulations.

Patients with histological diagnosis of B-AS were included into the study. Each center reviewed the pathological samples for the purpose of the study and confirmed the diagnosis. To confirm the morphological diagnosis, the standard immunohistochemical (IHC) panel consists of the following markers: ERG and CD31 (endothelial markers), cytokeratin AE1, and CAMTA1 and TFE3 (to rule out epithelioid hemangioendothelioma)^15^.

The data collection included demography, clinical presentation, type of treatment (local and systemic) and outcome. In cases with full pathology report available IHC markers were also reported.

Depending on the disease presentation, patients were classified as localized or metastatic (any site). The primary tumor locations were grouped as follows: extremity, pelvis/sacrum and central (all axial lesions excluding pelvis/sacrum). Surgical margins were considered adequate if wide or radical, and inadequate when intralesional, marginal or contaminated^16^. A surgical complete remission (SCR) was defined as the surgical removal of the primary tumor and, for metastatic patients, of all sites of metastatic disease. The response to chemotherapy (when available) in metastatic patients was assessed according to RECIST 1.1^17^.

Anonymized data has been sent to the study-referral center (IRCCS Istituto Ortopedico Rizzoli, Bologna, Italy) that performed the statistical analysis. Descriptive statistics were used to summarize population characteristics. Comparisons between groups were made by chi-square analysis or using Fisher’s exact test when appropriate.

Overall survival (OS) and disease-free survival (DFS) were estimated using the Kaplan-Meier analysis. OS was calculated from the date of diagnosis to death (any cause) or to last follow-up.

DFS was calculated from date of SCR to the occurrence of local or distant recurrence, death of any cause or last follow up.

OS and DFS were analyzed with respect to potentially prognostic variables including gender, age (≤ 50 years,> 50 years), stage (localized vs. metastatic), administration of chemotherapy and/or radiotherapy, and achievement of a SCR status. Multivariate analysis including significant and clinically relevant variables was performed.

## Results

### Demographic

Nine centers (IRCCS Istituto Ortopedico Rizzoli, Bologna, Italy; Department of Orthopaedics and Trauma, Medical University of Graz, Austria; Medical University of Vienna, Department of Orthopaedic Surgery, Vienna, Austria; Westfalian Wilhelms University, University Hospital Muenster, Department of Orthopaedics and Tumor Orthopaedics, Muenster, Germany; Department of Oncology and Department of Orthopaedic Surgery, Oslo University Hospital, Oslo, Norway; Bone Tumour Unit, Department of Orthopaedics and Traumatology Helsinki University Hospital, Helsinki, Finland; Department of Orthopaedic Surgery Eberhard Karls University, Tuebingen, Germany; Department of Orthopaedic Surgery, University Medical Center Groningen, University of Groningen, the Netherlands; Orthopedic University Hospital University of Coimbra, Coimbra, Portugal) with a specific expertise in sarcomas from 7 European countries participated in the study.

Data from 89 patients treated between 1976 and 2017 were collected. Six patients were excluded due to incorrect diagnosis and three patients had missing data; 80 patients were included in the present analysis.

The clinical characteristics of the patients are reported in Table 1, with IHC markers in a subset of the cases summarized in Table 2. The study population comprised patients with a wide range of age with a prevalence of male gender. Femur and pelvis were the most frequent primary tumor locations. The incidence of patients with metastatic disease at diagnosis was 44%. Multivisceral metastases were identified in 16 patients: lung metastases in seven patients, splenic in one patient, lymph node in one with eight patients had multifocal bone locations, and the site was not specified in two patients. There were no differences in clinical presentation according to age, sex and site between localized and metastatic patients (Table 1). The IHC panel confirms that both presence of endothelial markers (CD31 and ERG) and lack of TFE3 and CAMTA have high specificity and sensibility for the diagnosis of angiosarcoma of bone, with focal expression of cytokeratin AE1/AE3 in 9% of the cases (Table 2, Fig. 1).

**Table 1:** Clinical Characteristics.

	All	Metastatic 35(44) n (%)	Localized 45(56) n (%)	p
**Sex**				0.7
F	29 (36)	12 (34)	17 (38)	
M	51 (64)	23 (66)	28 (62)	
**Site of primary**				0.4
Extremity	45 (56)	18 (52)	27 (60)	
Central	16 (20)	6 (17)	10 (22)	
Pelvis + Sacrum	19 (24)	11 (31)	8 (18)	
**Age**				0.9
median (min-max)	54 (17–92)	58 (23–92)	53 (17–74)	
≤ 50 yrs	28 (35)	12 (34)	16 (36)	
> 50 yrs	52 (65)	23 (66)	29 (64)	
**Pattern of metastases**				na
Multiple sites	16 (46)	16 (46)	na	
Bone (multifocal)	8 (23)	8 (23)	na	
Lungs	7 (20)	7 (20)	na	
Nodes	1 (3)	1 (3)		
Spleen	1 (3)	1 (3)		
Unknown	2 (5)	2 (5)		

na = not applicable.

**Table 2:** Immunohistochemical angiosarcoma tumor markers expression.

Antibody	Percent of positive tumors	
	n	%
CD31	47	100
ERG	47	100
Cytokeratin AE1	4	9
TFE3	47	0
CAMTA1	47	0

### Local treatment

#### Surgery

Sixty-one patients (76%) out of 80 underwent surgery of the primary lesion: 87% (39 of 45) in patients with localized disease and 36% (22 of 35) in cases with metastatic disease. Eighteen (30%) patients had an amputation, while 43 (70%) patients underwent other surgical procedures.

Surgical margins, available in 38 cases, were adequate in 24 (63%), and inadequate in 14 (37%) patients (10 intralesional and four marginal). Focusing on localized patients, surgical margins were reported in 26 patients and were wide in 19, marginal in two and intralesional in five patients.

Overall, data on surgical complete remission (SCR) were available for 76 patients. The rate of SCR was 47% (one of the 35 patients presenting with metastases achieved SCR, and 35 of 45 patients with localized disease).

### Radiotherapy

Data on the use of radiotherapy were available in 77 patients: 32 received radiotherapy: two (6%) patients, both presenting with metastases, received radiotherapy as definitive local treatment, eight (25%) patients with localized disease underwent adjuvant radiotherapy after surgery, and in the remaining 22 (69%) patients low dose (about 30 Gy) radiotherapy was administered with palliative intent.

### Chemotherapy

Data on chemotherapy use were available in 77 patients. Chemotherapy was administered to 36 (47%) patients, 18 had localized disease and 18 had synchronous metastases.

Adjuvant regimens included osteosarcoma-like treatment (doxorubicin, methotrexate, cisplatin and ifosfamide) in six patients (33%), doxorubicin ± ifosfamide in four (22%), three (17%) and two (11%) patients respectively underwent paclitaxel and gemcitabine, while the type of treatment was not reported in three cases. For metastatic patients chemotherapy regimens adopted in first line and RECIST responses are detailed in Table 3.

**Table 3:** Best response in 18 patients with metastatic disease receiving chemotherapy (RECIST 1.1).

Regimen	n° (%) of pts	Best Result (RECIST 1.1)		
		PR	SD	PD
Doxorubicin- Ifosfamide	8 (44)		2	6
Osteosarcoma-like	4 (22)		1	3
Paclitaxel	3 (17)	1		2
Gemcitabine	2 (11)		2	
Caelix	1 (6)			1

^*^Standard doxorubicin or pegylated liposomal doxorubicin.

### Outcome

With a median follow-up of 31 months (from 4 to 309 months) 54 (68%) patients were dead of disease, 23 (16%) were alive with no evidence of disease, 10 (12%) were alive with disease and 3 were dead of other causes (thyroid cancer, colon cancer, not specified in one case).

**Figure 2:**
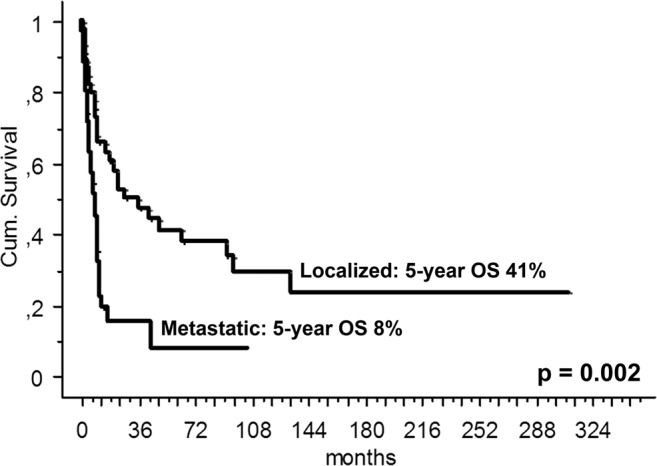
Five-year overall survival (OS) of 80 patients with bone angiosarcoma by stage at presentation.

The 5-year OS was 27% (95%CI 16–30), 41% (95%CI 25–56) for localized and 8% (95%CI 0–20) for metastatic patients (p = 0.002) (Fig. 2).

### Patients with localized disease

The 5-year OS was 45% (95%CI 28–62) for 39/45 patients with localized disease who underwent surgery and achieved a SCR status and 17% (95%CI 0–46) for 6/45 patients not surgically treated (p < 0.001).

Limiting the analysis to 26 patients with localized lesions and available information on quality of margins, 5-year OS was 42% for patients with wide margins. None of the patients with intralesional margins were alive at 5 years and only one of the two cases with marginal margins was alive at the 5-year mark.

In six patients with localised disease who did not undergo surgery, the tumor was mainly located in the axial skeleton (four pelvis/sacrum, one spine, and one femur). In five of the patients exclusive radiotherapy was performed. All patients with no surgery have died with a median time to death of nine months (range 3–27 months), while one patient undergoing gemcitabine after radiation therapy on the primary tumor was alive with disease after 29 months of follow-up.

**Figure 3:**
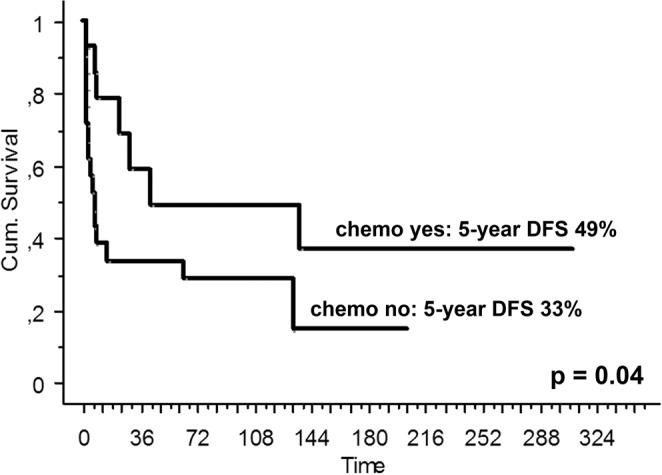
Five-year diseases-free survival (DFS) in patients with localized disease and who achieved a surgical complete remission (SCR), according to the use of adjuvant chemotherapy.

The 5-year DFS was 37% (95% CI 19–56), better in patients ≤ 50 years old), and in those who received adjuvant chemotherapy (Table 4; Fig. 3). Four of the five patients (80%) with localized disease and SCR who received adjuvant radiotherapy were free of disease at the 5-year timepoint, compared with 34% in those who did not.

**Table 4:** Univariate analysis for disease-free survival (DFS) in localised and surgically treated patients and complete remission (SCR).

Variable		Pts n°	5-year DFS	95% CI	p
All		35	37	19–56	
Age	≤ 50 yrs	12	74	49–100	0.0007
	> 50 yrs	23	22	4–41	
Sex	M	23	43	23–64	0.9
	F	12	29	0–61	
Site	extremity	25	46	26–66	0.1
	central	6	33	0–71	
	pelvis+sacrum	4	25	0–67	
Chemotherapy	Yes	14	49	19–78	0.04
	No	21	33	13–53	
Radiotherapy*	Yes	5	80	45–100	0.2
	No	29	34	16–53	

M: male; F: Female; DFS: disease-free survival.

*Radiotherapy information was available for 34 patients.

The multivariate analysis demonstrated that patients who were less than 50 years of age and use of radiotherapy were associated with improved DFS in patients with localized tumors able to achieve SCR (Table 5).

**Table 5:** Multivariate analysis for disease-free survival (DFS) in localised and surgically treated patients and complete remission (SCR).

Variable		RR	95% CI	p
Age	> 50 yrs	1		0.01
	≤ 50 yrs	0.17	0.04–0.68	
Site	pelvis+sacrum	1		0.96 0.21
	central	1.04	0.16–6.67	
	extremity	0.43	0.11–1.63	
Chemotherapy	Yes	1		0.18
	No	1.97	0.73–5.36	
Radiotherapy*	Yes	1		0.04
	No	6.4	1.08–37.97	

RR: relative risk; CI: confidence interval; M: male; F: Female.

*Radiotherapy information was available for 34 patients.

After SCR, a better OS was reported in younger patients (≤ 50 years old) and in those patients with tumors located in the extremities, none confirmed at multivariate analysis (Tables 6, 7).

**Table 6:** Univariate analysis for overall survival (OS) in localised and surgically treated patients and complete remission (SCR).

Variable		Pts n°	5-year 0S	95% CI	p
All		39	45	28–62	
Age	≤ 50 yrs	13	68	41–94	0.02
	> 50 yrs	26	34	13–55	
Sex	M	26	57	26–77	0.3
	F	13	25	0–53	
Site	extremity	26	50	29–72	0.008
	central	9	58	22–95	
	pelvis+sacrum	4	0		
Chemotherapy*	yes	16	49	19–78	0.4
	no	21	42	20–63	
Radiotherapy**	yes	8	71	38–100	0.4
	no	30	40	20–60	

OS: overall survival; CI: confidence interval; M: male; F: Female.

*Chemotherapy information was available for 37 patients; **Radiotherapy information was available for 38 patients.

**Table 7:** Multivariate analysis for overall survival (OS) in localised and surgically treated patients and complete remission (SCR).

Variable		RR	95% CI	p
Age	> 50 yrs	1		0.08
	≤ 50 yrs	0.35	0.1–1.1	
Site	pelvis+sacrum	1		0.2 0.04
	central	0.3	0.1–1.9	
	extremity	0.25	0.1–0.9	
Chemotherapy*	yes	1		0.9
	no	0.9	0.3–2.5	
Radiotherapy	Yes	1		0.5
	No	1.5	0.4–6.2	

RR: relative risk; CI: confidence interval.

*Chemotherapy information was available for 37 patients; **Radiotherapy information was available for 38 patients.

### Patients with synchronous metastases

Of the 35 patients with synchronous metastases, 29 have died, with a median time to death of six months (range 1–49 months), six patients were alive with disease, with a median time of observation of 22 months (range 8–106 months). One of them was 39-year old male with a B-AS localized in the pelvis and multiple metastases. He underwent chemotherapy with doxorubicin and ifosfamide, radiotherapy on primary tumor, lung and bone metastases, and several surgical treatments in the ileum and in the hip for pathological fractures. He was still alive 9 years after diagnosis.

The 1- year OS was 22% (95% CI 8–37) and the 5-years OS was 8% (95% CI 0–20). Only one patient with angiosarcoma of the femur and lung metastases achieved a SCR status. After surgical resection of the primary tumor and metastases, he underwent adjuvant radiotherapy and systemic chemotherapy treatment. He died of disease (brain metastases) 12 months after diagnosis.

The OS was not influenced by the use of chemotherapy: the 2-year OS was 15% [95% CI 3–43] in 18 patients who underwent chemotherapy and 17% [95%CI 0–38] in those (n = 14) who did not. A partial response (PR) was documented in one patient who received paclitaxel, while stable disease (SD) was reported in five patients (two after gemcitabine, two after doxorubicin and ifosfamide and one after a osteosarcoma-like regimen) (Table 3). The 1-year OS was 67% (95CI% 29–100) for those who responded or had stable disease (n = 7), and 18% (95CI% 0–41) for patients with progressive disease (n = 11), p 0.002. Metastatic pattern and age did not influence the survival rates.

## Discussion

This study, carried out in the framework of the EMSOS network, confirms the rarity and the aggressive behaviour of primary B-AS. The scarcity of the patient population represents a treatment challenge, even in referral centers.

Most of the patients were metastatic at diagnosis (44%), and this incidence is twice that reported in other bone sarcomas^18^. Furthermore, half of the patients presented with metastases in multiple sites (only 9% of the patient had lung only metastases).

Overall the prognosis of B-AS is very poor, with a 5-year survival rate for metastatic patients of only 8%, and also poor results also for patients with localized disease unable to achieve a SCR status (5-year OS 17%). Still, it is important to underscore that for a small subgroup of patients (12 patients with localized angiosarcoma younger than 50 years of age) the 5-year OS was 68%, not much different from high-grade bone tumors, such as osteosarcoma.

This heterogeneity in clinical behaviour might be related to the high genetic variability described in angiosarcomas, with several genes abnormalities identified in B-AS patients such as CIC, PLCG1, KDR and MYC (14–15).

The main factors significantly related to the survival were the stage, patient age and the site of the tumor.

Overall, the local treatment of these tumors reflects their aggressiveness: In about 1/4 of patients surgery of the primary tumor was not feasible, and when surgery was performed, in 30% of patients it was an amputation.

Our study confirms the importance of surgery in patients with localized disease, showing a survival benefit in those patients who achieved a SCR status. Achieving adequate surgical margins is important, as no patients with an intralesional resection survived. Therefore a surgical approach should only be considered if it is likely achieve adequate surgical margins.”

In metastatic patients, it is questionable to adopt aggressive surgical procedures since the low, if any, possibilities of achieving a surgical remission. Furthermore, it is important to note that the only one metastatic patient who was rendered free of disease, soon relapsed with distant metastases.

No definitive conclusions can be drawn regarding the role of chemotherapy on B-AS. The data collected in the present analysis indicates that chemotherapy might be of assistance in B-AS. In metastatic patients overall the use of chemotherapy did not offer a better survival rate, but it is relevant to note that a significantly better 1-year OS was observed in the few patients who had a disease stabilization after chemotherapy. A significantly better DFS was reported, in localized patients who achieved a complete SCR and received adjuvant chemotherapy. This was not confirmed after multivariate analysis. On the other hands, this is a small sample size and these results should be taken with some caution due to the small number of patients considered.

Overall, the data reported both on adjuvant chemotherapy advantage in patients with localized B-AS achieving a SCR status and the evidence of tumor shrinkage and/or disease stabilization with taxol and gemcitabine in some patients suggests that chemotherapy has some activity in B-AS; nonetheless at present no recommendation on superiority of a specific chemotherapy regimen can be provided and more effective systemic treatment are needed.

Radiotherapy was mainly used with palliative intent in metastatic patients and in localized patients when surgery was not feasible. Interestingly, 4 of the 5 patients with localized disease and SCR who received adjuvant radiotherapy were free of the disease after 5 years. With the limitation of the small number of patients, the multivariate analysis does show an advantage from adjuvant radiotherapy in B-AS with SCR. It will be important in future studies to address whether the current radiotherapy techniques, including protontherapy, or tomotherapy can offer some advantages in terms of tumor control.

Multivariate analysis has a number of limitations owing to the small number of patients included in this study. Nonetheless, it confirms a better prognosis in terms of DFS for patients younger than 50 years old and it shows that radiotherapy might reduce the risk of relapse for patients with localised B-AS achieving surgical complete remission.

## Conclusions

B-AS is a rare and aggressive tumor with heterogeneous behaviour. Overall, patients with metastatic B-AS have a dismal prognosis, with prolonged survival in cases that respond to chemotherapy. Experimental trials focusing on more active systemic treatment regimens are needed.

In patients with localized disease, the patient’s age and site of the tumor are prognostic factors and any effort must be made to achieve a SCR status. No definitive conclusions can be drawn from our data on the use of adjuvant chemotherapy while the use of adjuvant radiotherapy might improve DSF in localized patients surgically free of disease.
